# Updates on malaria epidemiology and profile in Cabo Verde from 2010 to 2019: the goal of elimination

**DOI:** 10.1186/s12936-020-03455-7

**Published:** 2020-10-23

**Authors:** Adilson José DePina, Gillian Stresman, Helga Sofia Baptista Barros, António Lima Moreira, Abdoulaye Kane Dia, Ullardina Domingos Furtado, Ousmane Faye, Ibrahima Seck, El Hadji Amadou Niang

**Affiliations:** 1 Programa de Eliminação do Paludismo, CCS-SIDA, Ministério da Saúde e da Segurança Social, Praia, Cabo Verde; 2 Ecole Doctorale des Sciences de la Vie, de la Santé et de l’Environnement (ED-SEV), Université Cheikh Anta Diop (UCAD) de Dakar, Dakar, Sénégal; 3 Faculty of Infectious & Tropical Diseases, London School of Hygiene & Tropical Medicine, London, UK; 4 Delegacia de Saúde da Praia, Praia, Cabo Verde; 5 Programa Nacional de Luta contra as Doenças de Transmissão vectorial e Problemas de Saúde Associadas ao Meio Ambiente, Ministério da Saúde e da Segurança Social, Praia, Cabo Verde; 6 Laboratoire d’Ecologie Vectorielle et Parasitaire,Faculté des Sciences et Techniques, Université Cheikh Anta Diop (UCAD) de Dakar, Dakar, Sénégal; 7 Institut de Santé et Développement, Université Cheikh Anta Diop (UCAD) de Dakar, Dakar, Sénégal; 8 Aix Marseille Univ, IRD, AP-HM, MEPHI, IHU-Méditerranée Infection, Marseille, France

**Keywords:** Surveillance, Imported infections, Prevention of reintroduction

## Abstract

**Background:**

Located in West Africa, Cabo Verde is an archipelago consisting of nine inhabited islands. Malaria has been endemic since the settlement of the islands during the sixteenth century and is poised to achieve malaria elimination in January 2021. The aim of this research is to characterize the trends in malaria cases from 2010 to 2019 in Cabo Verde as the country transitions from endemic transmission to elimination and prevention of reintroduction phases.

**Methods:**

All confirmed malaria cases reported to the Ministry of Health between 2010 and 2019 were extracted from the passive malaria surveillance system. Individual-level data available included age, gender, municipality of residence, and the self-reported countries visited if travelled within the past 30 days, therby classified as imported. Trends in reported cases were visualized and multivariable logistic regression used to assess risk factors associated with a malaria case being imported and differences over time.

**Results:**

A total of 814 incident malaria cases were reported in the country between 2010 and 2019, the majority of which were *Plasmodium falciparum*. Overall, prior to 2017, when the epidemic occurred, 58.1% (95% CI 53.6–64.6) of infections were classified as imported, whereas during the post-epidemic period, 93.3% (95% CI 86.9–99.7) were imported. The last locally acquired case was reported in January 2018. Imported malaria cases were more likely to be 25–40 years old (AOR: 15.1, 95% CI 5.9–39.2) compared to those under 15 years of age and more likely during the post-epidemic period (AOR: 56.1; 95% CI 13.9–225.5) and most likely to be reported on Sao Vicente Island (AOR = 4256.9, 95% CI = 260–6.9e+4) compared to Boavista.

**Conclusions:**

Cabo Verde has made substantial gains in reducing malaria burden in the country over the past decade and are poised to achieve elimination in 2021. However, the high mobility between the islands and continental Africa, where malaria is still highly endemic, means there is a constant risk of malaria reintroduction. Characterization of imported cases provides useful insight for programme and enables better evidence-based decision-making to ensure malaria elimination can be sustained.

## Background

Malaria elimination means, a country or territory, has had at least three consecutive years without any indigenous case of malaria, and can apply for certification from the World Health Organization (WHO) to be listed among the "malaria-free countries" [1]. To achieve malaria elimination, it requires an unremitting political commitment, substantial and predictable financing and increased regional collaboration [2]. To fuel the drive for malaria elimination, the WHO identified 21 countries in five regions, which could overcome malaria by 2020, a process that accounted for both technical, operational, and biological factors [2]. In according with WHO data, since 2016, 49 countries reported fewer than 10,000 cases, five countries have reported no infections since 2017, placing them on track for malaria elimination certification, and five of others have received malaria elimination certification [3–8].

Cabo Verde is one of 21 countries identified with the potential to achieve malaria elimination by 2020 [2]. Located off the coast of West Africa, malaria has been endemic in the country since the colonization period [9]. Historically, malaria transmission was low, with sporadic epidemics linked with unseasonably high rainfall [10]. Malaria transmission has been interrupted twice in the country’s history but has not been sustained, likely due to a high degree of population movement between the islands and endemic countries [11]. In recent years, malaria transmission in Cabo Verde has been low and unstable, with sporadic and seasonal transmission associated with the rainy season and increasing vector densities [12–14].

Cabo Verde is again in a position where endemic malaria transmission in the country has been interrupted and will be eligible for applying for WHO certification in January 2021. To ensure elimination can be sustained and to support the prevention to reintroduction phase, the malaria case data collected as part of routine surveillance from the decade before interruption can be valuable for characterizing the malaria epidemiology and identify risk factors for improved and targeted surveillance activities. Therefore, this work extends previous research characterizing malaria cases in the country [13, 14] and describes both individual and spatial risk factors for endemic and introduced malaria infections.

## Methods

### Study area

**Fig. 1:**
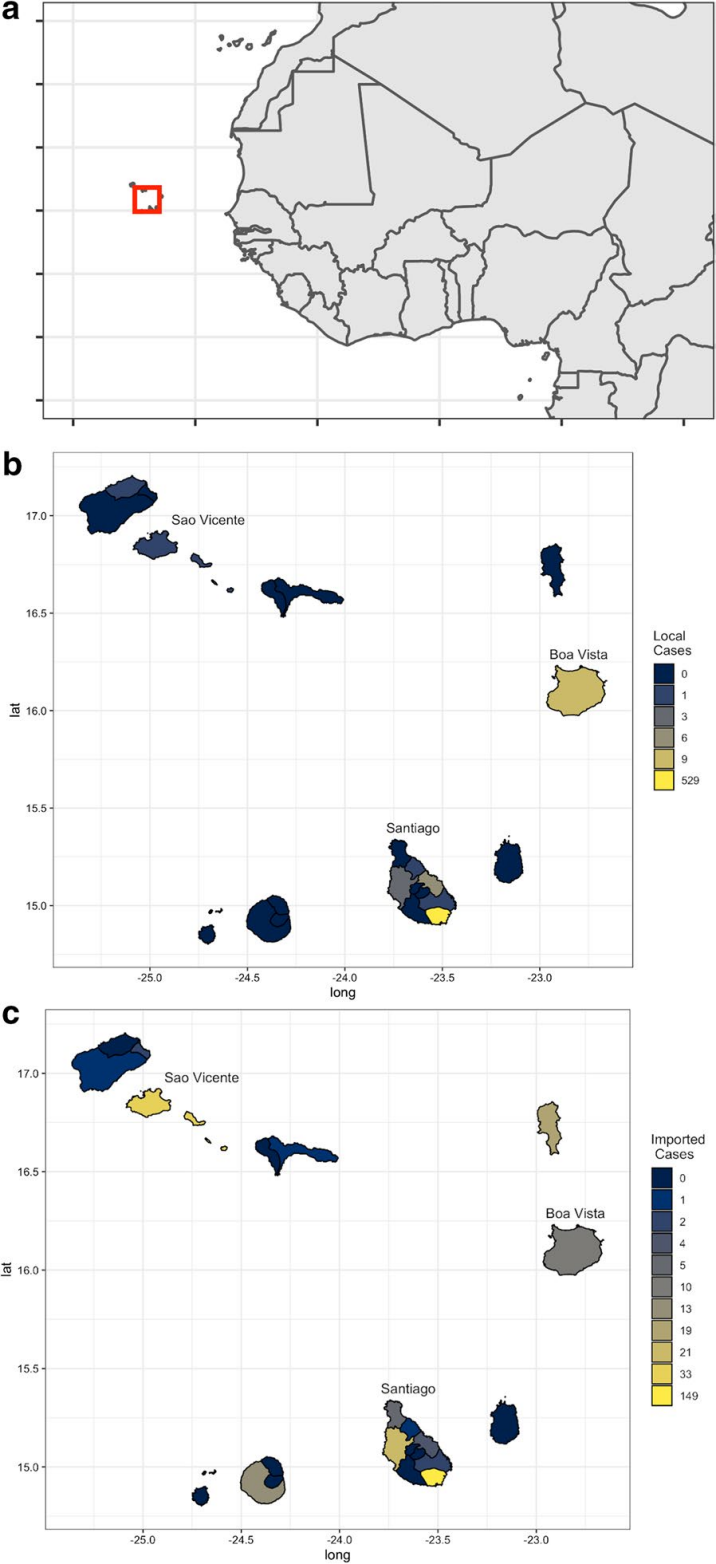
The location of Cabo Verde (**a**) shown in red off the coast of Senegal, in reference to continental Africa. Total number of locally acquired (**b**) and imported malaria cases (**c**) in Cabo Verde between 2010 and 2019 by municipality

Cabo Verde is an archipelago of volcanic origin located approximately 450 km from the West African coast, west of Dakar, Senegal with approximately 550,000 residents (2019 estimates) [15] (Fig. 1a). It occupies an area of 4033 km^2^ and is composed of ten islands, nine of which are inhabited. The islands are composed of a hilly terrain, with only about 10% of the land available for farming. The temperature in the country ranges from a maximum of 25 ºC to 30 ºC from August to October, with the lowest temperatures around 19 ºC to 25 ºC, typically from January to February. Like all Sahelian zones, the archipelago has a contrasting wet and dry season. The rain is irregular and the archipelago undergoes periodic droughts. The average annual precipitation ranges between 300 and 700 mm in the low-lying and high-altitude areas, respectively [16].

Administratively, the country has 22 municipalities and 24 cities. The health system consists of two national reference hospitals (Agostinho Neto in Praia, Santiago Island and Baptista de Sousa in Mindelo, São Vicente), four regional hospitals (in Santo Antão, Sal, Fogo and Santa Catarina in Santiago), and 30 health centres. There are also 3 specific centres, 34 health posts that work with nurse oversight and 113 Basic Health Units and two Health Delegations. Access to healthcare is free for all residents and malaria is a notifiable disease, an essential component of the elimination programme. All suspected malaria cases are tested by rapid diagnostic test and confirmed by microscopy (see Table 1 for case definition applied in Cabo Verde [17, 18]). All confirmed cases are treated as inpatients and reported to the National Malaria Control Programme (NMCP) and the Integrated Surveillance and Response to Epidemics (SVIRE) programme within 24 h, prompting an immediate foci investigation. Age, gender, test result, residential address and travel history, used to classify cases as indigenous or imported (Table 1), are recorded for each confirmed case. The number of people tested or suspected for malaria was not available for this analysis. The five infections classified as being relapses were removed from this analysis as they did not represent incident infections.

**Table 1:** Case definition and classification for malaria used in Cabo Verde and consistent with the WHO definitions [17, 18]

Case definition	
Malaria case, confirmed	Malaria case (or infection) in which the parasite has been detected in a diagnostic test, i.e., microscopy, a rapid test or a molecular diagnostic test
Malaria, all cases	All malaria cases irrespective of Plasmodium species (including imported case)
Malaria case, imported	Malaria case or infection in which the infection was acquired outside the area in which it is diagnosed (get infected outsider from Cabo Verde)
Malaria case, indigenous	A case contracted locally with no evidence of importation and no direct link to transmission from an imported case (get infected in Cabo Verde)
Malaria elimination	Interruption of local transmission (reduction to zero incidence of indigenous case) of a specified malaria parasite in a defined geographical area as a result of deliberate activities. Continued measures to prevent re-establishment of transmission are required
Case classification	
Imported case	Malaria case or infection in which the infection was acquired outside the area in which it is diagnosed. Infection from a country outside of Cabo Verde
Indigenous case	A case contracted locally with no evidence of importation and no direct link to transmission from an imported case
Introduced case	A case contracted locally, with strong epidemiological evidence linking it directly to a known imported case (first-generation local transmission)
Recrudescent case	Malaria case attributed to the recurrence of asexual parasitaemia after anti-malarial treatment, due to incomplete clearance of asexual parasitaemia of the same genotype(s) that caused the original illness

The individual-level malaria case data reported from January 2010 to December 2019 was obtained from the Ministry of Health. All data are anonymous and collected as part of routine public health activities so ethical approvals or individual consent were not sought. However, permission for use of this data was obtained by the Malaria Elimination Programme.

### Data analysis

Descriptive statistics and visualisation were used to show the variation of malaria cases during the period, stratified by indigenous and imported, pre/post epidemic period, age, gender (as % male), and island. Annual incidence per 1000 and mortality per 100,000 rates were calculated using the annual population estimate for Cabo Verde per year [15]. The Case fatality rate (CFR) was also determined according to the number of deaths and confirmed cases reported per year.

Multivariable logistic regression was applied to assess risk factors associated with imported malaria in Cabo Verde. Analysis was conducted in R statistical software (V 3.6.1). Covariables available for the risk factor analysis included time period, categorized as pre-epidemic (2010–2016), epidemic (2017) and post-epidemic (2018–2019) period, the island where the case was reported aggregated based on the number of reported cases (Boavista, Santiago, Sao Vicente, and Other), age, and gender. The logged total population residing on the island was included as a continuous variable to account for differential probabilities of detection in large compared to small populations. Interactions between year and epidemic period, and gender and age category were also tested. The best model fit was ascertained uing the AIC and followed a backwards model selection process. To assess the likelihood of achiving malaria elimination, the annual estimate of the basic reproductive number (R_0_) was calculated according to the ratio of local to imported infections using methods developed by Churcher et al*.* [19].

## Results

### Number of malaria cases

**Fig. 2:**
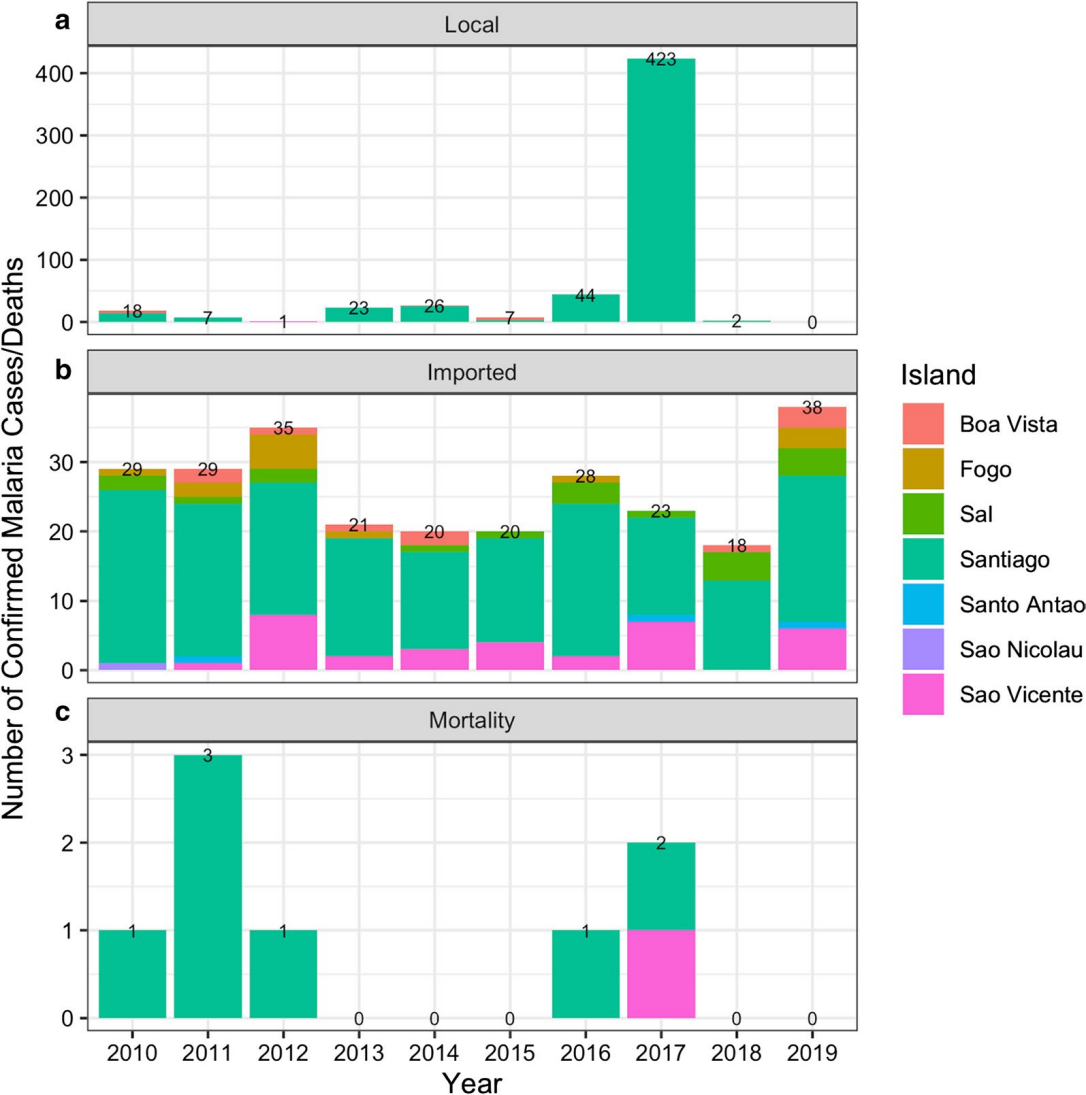
The total number of malaria cases (**a**), imported infections (**b**) and deaths (**c**) per year. The different colours within each bar represent the number of events reported by each island, with each bar labelled with the total number of cases per year

Over the ten year period between 2010 and 2019, 814 confirmed incident malaria cases were reported in Cabo Verde, of which 554 (67.6%) were classified as indigenous and 263 (32.1%) as imported. There also were two as introduced cases, one each in 2018 and 2019. However, 423 (76.4%) of the locally acquired cases occurred during the outbreak between July to October, 2017, representing 94.8% (423/446) of all cases in that year (Table 2). Imported cases have been reported from almost all municipalities and the last local case was reported in January 2018 (Figs. 1b, 2a). There were 0 to 30 cases reported per month with imported infections consisting of 58.1% of all confirmed cases in the pre-epidemic, and between 0 and 1 case reported with 93.3% of cases in the post-epidemic period being imported (Table 2). Both locally acquired and imported infections occur in the majority of municipalities across the country (Figs. 1c, 2b). Malaria incidence per 1000 populations was estimated to be less than 0.1 for all years except for during the 2017 epidemic (0.8/1000 people). Malaria mortality was very low throughout the study period, with a total of nine deaths (range 0 to 3; the maximum case fatality rate was 8.3%) and a corresponding mortality rate ranging from 0 to 0.6 per 100,000 populations at risk, per year (Fig. 2c).

**Table 2:** Demographic characteristics of confirmed malaria infections reported in Cabe Verde between 2010 and 2019, stratified by pre-epidemic, epidemic, and post-epidemic years as well as locally acquired and imported infections as classified by routine malaria programs

	Pre-epidemic years (2010–2016)		Epidemic (2017)		Post-epidemic (2018–2019)	
	Malaria cases (N = 308)	95% CI	Malaria cases (N = 446)	95% CI	Malaria cases (N = 60)	95% CI
Imported infections—% (n)	58.1 (182/308)	53.6–64.6	5.2 (23/446)	3.0–7	93.3 (56/60)	86.9–99.7
Island—% of infections imported (n/N)						
Boavista	40.0 (6/15)	14.3–65.7	0	–	100 (4/4)	–
Santiago	53.6 (134/250)	47.4–59.8	3.2 (14/437)	1.2–4.9	89.5 (34/38)	79.6–99.4
Sao Vicente	95.2 (20/21)	85.9–100	100 (7/7)	–	100 (6/6)	–
Other	100 (22/22)	–	100 (2/2)	–	100 (12/12)	–
Gender—% of all infections in males (n/N)						
Imported	82.1 (147/182)	76.5—87.7	78.3 (18/23)	61.0–95.5	75.0 (3/4)	69.9–90.9
Locally acquired	73.8 (93/126)	66.1–81.5	69.50 (294/423)	65.1–73.9	80.4 (45/56)	26.0–100
Median age (IQR)	33.0 (25.0–42.0)	–	30.0 (20.0–43.0)	–	35.5 (27.0–41.25)	–
Locally acquired	28.0 (17.3–40.8)	–	30.0 (20.0–43.0)	–	14.5 (8.8–25.0)	–
Imported	35.0 (28.0–46.0)	–	33.0 (31.0–39.5)	–	36.0 (28.0–41.8)	–

The total (N), percentages and corresponding 95 Confdience Intervals (95% CI) are included

### Demographic characteristics of malaria cases

Overall, there was good routine reporting of demographic information with only two individuals missing the gender classification and 16 missing age. Across the ten-year period, 73.7% (600/814) of all cases were male, and was a consistent between imported and locally acquired infections (Table 2). Similarly, the median age of cases was 33 years (IQR = 20–43 years). However, individuals with imported malaria were older compared to those with locally acquired infections, particularly in the post-epidemic period with median age of cases of 36 and 14.5, respectively. Locally acquired cases were consistently reported between August and October (75.4%) whereas imported infections were reported throughout the year, ranging from 3.4% in February to 13.3% of infections in October. However, the highest risk period for imported infections was between August and January, with 64.6% of all imported infections reported, coinciding with the main transmission period in west African countries.

**Fig. 3:**
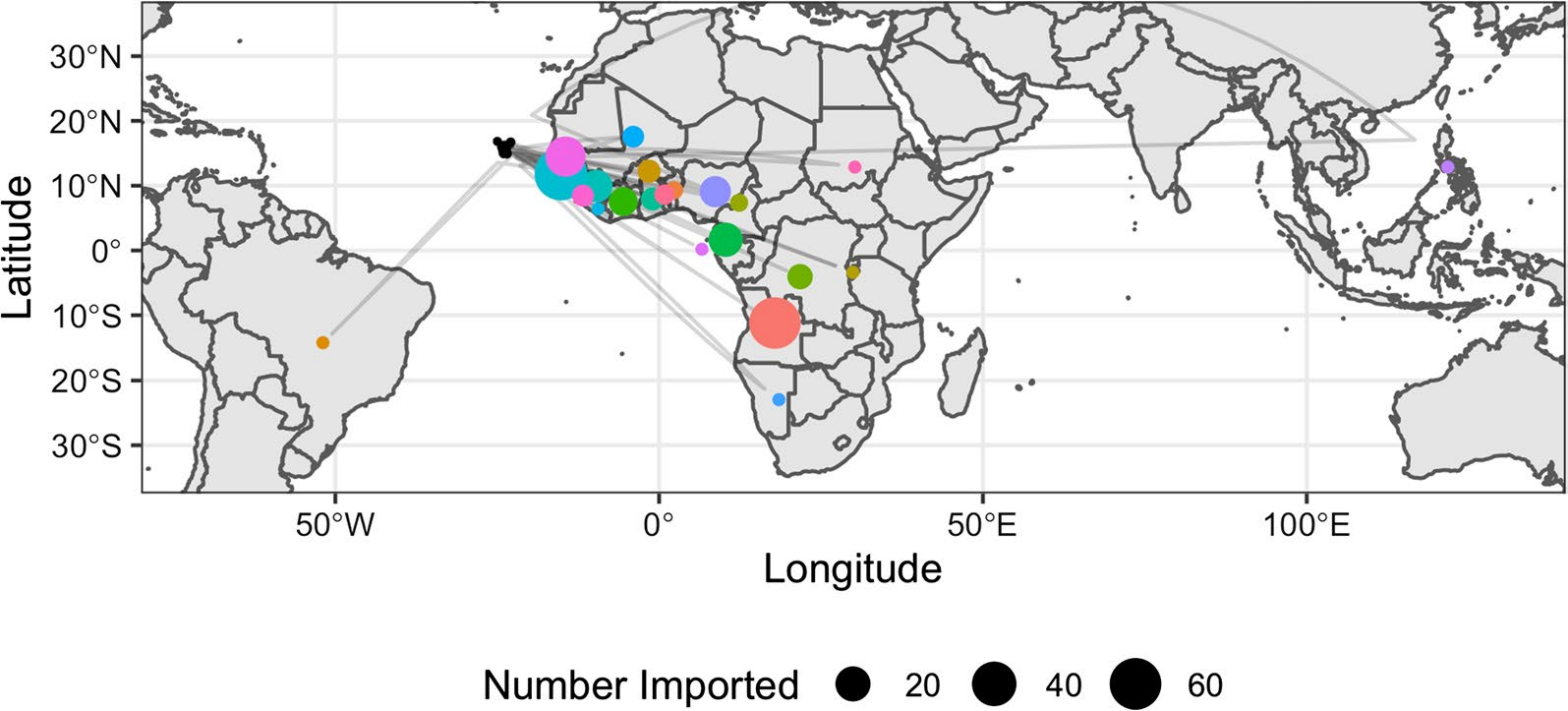
Global map showing the likely origin of imported infections that were reported in Cabo Verde between 2010 and 2019. The size of the circle is scaled according to the number of cases likely originating in that country, with the different colours shown to differentiate the different countries. The location of Cabo Verde is shown as the black circles and connector lines shown in light grey

**Fig. 4:**
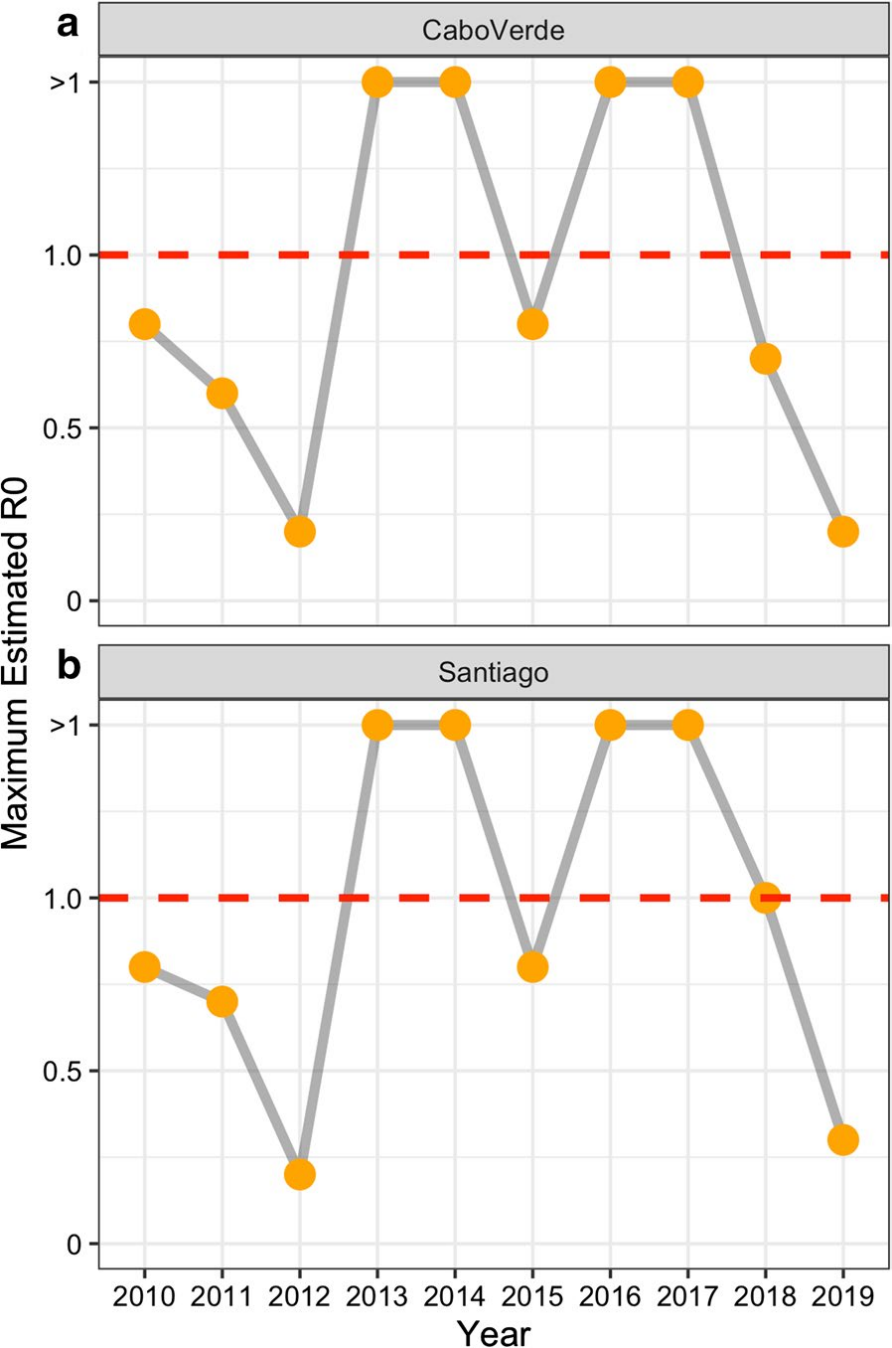
Estimated annual R0 according to the ratio of imported to local cases resported for Cabo Verde (**a**) and Santiago Island only (**b**) where there were sufficient cases (min 10 cases required for models) per year to obtain estimates. The y-axis presents the maximum estimate of R0 that is plausible based on the data with year presented on the x-axis. The red dashed line shows where R0 equals 1 whereby above this line transmission is increasing and below, transmission is expected to die out

Malaria cases were reported from the majority of municipalities in the country (14/22) at some point during the study period (Fig. 1a). The majority of cases were reported in Praia, on Santiago Island, the capital of the country (680; 83.5%), followed by São Vicente (34; 4.2%), Sal (19; 2.3%) and Boavista (19; 2.3%). The municipalities reporting the greatest proportion of the 261 imported infections were Praia (149; 57.1%), São Vicente (33; 12.6%), and Santa Catarina (21; 8.0%) (Fig. 1b). The likely origin of imported infections based on travel history spanned 22 countries including Brazil (the single case of *P. vivax)*, Philippines, and multiple African countries (Fig. 3). The majority of imported malaria infections had reported travel to Portuguese speaking countries, including 24.3% and 22.4% of cases recently travelling to Guinea Bissau and Angola, respectively. Other main countries where imported infections reported traveling to include Senegal (30; 11.4%), Equatorial Guinea (20; 7.6%), Nigeria and Guinea Conakry (both with 15; 5.7%) and Cote d´Ivoire (10; 3.8). The ratio of imported to locally acquired infections suggests that the estimated R_0_ was likely below 1 in 6 of the 10 years of analysis (Fig. 4a). According to the specific estimates for Santiago, where there were sufficient cases to determine the island specific estimates, the pattern was similar, except in 2018 where it lingered around 1 for that year (Fig. 4b).

The risk of malaria cases in Cabo Verde being imported increased significantly in the post epidemic period. Specifically, as compared to the pre-epidemic period, the odds of an infection being imported during the epidemic year was (AOR) 0.1 (95% CI 0.0–0.2) whereas this increased to 56.1 odds (95% CI 13.9–225.5) of infections being imported in the post-epidemic period (Table 3). Sao Vicente (AOR: 4256.9; 95% CI 260–6.9e+4) and Santiago (AOR: 23.9; 95% CI 4.4–130.8) had a greater odds of reporting an imported malaria case, Compared to Boavista. Finally, imported infections were more likely in those aged 25–40 (AOR: 15.1; 95% CI 5.9–39.2) compard to those under 15 years of age (Table 3).

**Table 3:** Results of the univariable and mulultivariable logistic regression analysis to assess risk factors associated with imported malaria in Cabo Verde

	Univariable analysis			Multivariable analysis		
	OR	95% CI	P-value	AOR	95% CI	P-value
Epidemic period						
Pre-epidemic	1.0	–	–	1.0	–	–
Epidemic year	0.0	0.0–0.1	< 0.001	0.1	0.0–0.2	< 0.001
Post-epidemic	9.7	3.4–27.4	< 0.001	56.1	13.9–225.5	< 0.001
Year	0.7	− 0.7–0.8	< 0.001	0.8	0.7–0.9	< 0.001
Island						
Boavista	1.0	–	–	1.0	–	–
Santiago	0.0	0.1–0.8	0.010	23.9	4.4–130.8	< 0.001
Sao Vicente	29.7	3.3–263.7	0.002	4256.9	260–6.96e+4	< 0.001
Other	1.4e+7	0-inf*	0.967	2.5e+8	0-inf	0.972
Gender (M vs F)	1.8	1.3–2.6	0.002			
Age category						
< 15 years	1.0	–		1.0	–	–
15–24	2.4	1.1–4.9	0.0	4.3	1.5–12.5	0.007
25–40	6.9	3.7–13.2	< 0.001	15.1	5.9–39.2	< 0.001
> 40	4.7	2.4–8.9	< 0.001	1.01	3.9–26.2	< 0.001
log10 (pop size)	0.02	0.01–0.05	< 0.001	0.34	0.2–0.7	0.001

The odds ratios (OR) and adjusted odds ratios (AOR) are presented, respectively, with the corresponding 95% Confidence Intervals (CI) and P-value

^*^ inf mean that the upper bounds of the confidence intervals extend to infinity, or not defined due to the low number of imported infections reported in these settings

## Discussion

If Cabo Verde can remain diligent, the country will be eligible to apply for malaria elimination certification in early 2021. Here, was described the malaria cases reported in Cabo Verde over the previous 10 years to characterize the transition to successfully eliminating transmission. This work highlights two, non-mutually exclusive challenges for elimination countries to sustain their gains: the risk of epidemics and continued importation. Reported locally acquired malaria cases prior to the 2017 epidemic were generally low, ranging from 0 to 30 cases per month and the estimated R_0_ being less than 1 for most years. In contrast, infections have been consistently imported into Cabo Verde and are reported in almost all municipalities. Understanding these trends, where and when imported infections are most likely to occur, and their potential to lead to onward transmission will be essential to sustain elimination and avoid future malaria epidemics.

To achieve malaria elimination in Cabo Verde, there has been a strong political commitment and investment in the health system [20–22]: there is good coverage of the health system across the country and is freely accessible to all residents. Malaria has been considered a notifiable disease for some time, to ensure that all cases are confirmed, offered supervised treatment with an effective antimalarial drug, and investigated within 24 h of reporting. Although information on individuals suspected and tested for malaria would strengthen confidence that all infections are identified, the data available for this analysis was largely complete with few missing data points. The investment to ensure complete, high-quality surveillance data will enable evidence-based targeting of resources and rapid responses to eliminate and monitor for any future epidemics [23]. Similarly, as part of strengthening the surveillance system, intensive training on classifying imported infections is essential to ensure local transmission is adequately measured. Effective classification and recording of the likely origin of infection also provides crucial information as to if and where intervention activities can be targeted to those individuals and/or areas most at risk [24–27]. Although the majority of infections were identified in males, this variable was not retained in the adjusted analysis. This is likely due to the limited heterogeneity for detecting associations. However, the results suggest that the frequent travel from Cabo Verde to endemic countries in continental Africa and being an adult under 40 years of age appears be the most important risk for re-introduction of malaria, putting the island’s malaria-free status at risk in the future [28, 29].

From the data over the ten years examined in this analysis, the capital city, Praia, experienced the highest burden of malaria cases in the country, including locally acquired (largely driven by the epidemic), imported, and also the two introduced infections. Praia is the largest urban area in the country, has the highest population of people traveling, including nationals and immigrant workers, and has frequent flight connections to endemic countries [28, 30]. Combined with the presence of semi-permanent breeding sites, which allow the persistence of *Anopheles arabiensis* in several neighbourhoods of the city, conditions in the city are highly conducive to malaria transmission [31–33]. Maintaining accessibility to health facilities, clinical management capacity for malaria and continued vector control will be important factors to prevent reestablishment of malaria transmission in the city, as well as other municipalities with a high degree of importation.

There are some limitations to this analysis to note. Firstly, the data available was obtained as part of routine surveillance. Although individual level case data from 2010 was available with few missing records, the breadth of potential covariates was limited thereby precluding more in depth analysis. Also, several variables were included in the regression modelling that serve as proxies. For example, although the population size was correlated with island, it improved the model fit and likely serves as a proxy for the probability of a case being identified. Similarly, epidemic period and year are also correlated. Despite the correlation and attempted alternative modelling forms (i.e. interactions), one of the variables may be acting as a proxy for changes in the surveillance system or malaria control program or shifts in how importated infections were classified. Finally, this data analysis was conducted with data collected as part of the routine malaria surveillance system. Therefore, not all potential covariables were available for analysis and the uneven distribution of indigeneous and imported infections across the islands and over time may have reduced statistical power to detect meaningful differences as is observed in the wide confidence intervals. Despite these limitations, the results are nevertheless informative.

## Conclusions

As presented here, Cabo Verde has interrupted malaria transmission. However, for this achievement to be sustained, understanding where the country may be vulnerable to reinitiation of transmission will be critical for evidence-based decision-making. The sustained low levels of transmission, strong public health system as shown as part of the 2017 epidemic, and active malaria programs in the country indicate that the archipelago can maintain a malaria-free state [12, 14, 17]. Given the likelihood of continued malaria importation from continental African countries, the risk associated with imported described here will be helpful in prioritizing resources. Ultimately, to prevent reintroduction, the malaria programme in Cabo Verde must remain diligent and monitor malaria importation in a manner that accounts for seasonality of malaria receptivity and vulnerability, while ensuring a sustained investment in the healthcare system.

## Data Availability

The datasets used and/or analysed during the current study are available from the corresponding author on reasonable request.

## Abbreviations

CFR: Case fatality rate
ED-SEV: Ecole Doctorale des Sciences de la Vie, de la Santé et de l’Environnement
NMCP: National Malaria Control Programme
SVIRE: Integrated Surveillance and Response to Epidemics
UCAD: Université Cheikh Anta Diop
WHO: World Health Organization
